# Do we need a replacement medication for influenza with good efficacy?

**DOI:** 10.1186/s40199-014-0084-3

**Published:** 2014-12-16

**Authors:** Mahmoud Arastoo, Hamid Reza Khorram Khorshid

**Affiliations:** Pharmaceutical Sciences Research Center (PSRC), Tehran University of Medical Sciences, Tehran, Iran; Genetic Research Center, University of Social Welfare and Rehabilitation Sciences, Tehran, Iran

## Dear editor-in-chief

In the first quarter of 2014, Cochrane collaboration, the independent, non-profit and non-governmental organization reported that Tamiflu (with the generic name of Oseltamivir) is not working effectively [1]. Furthermore, it was causing side effects including vomiting, nausea, headache, renal problems, psychiatric events, and the risk of other serious complications such as pneumonia when used for the treatment and prophylaxis of influenza. In addition, it may induce serious heart rhythm problems as the drugs regulator claims. The medicine was given to millions of people during the swine flu pandemic. However, Roche the Swiss drug manufacturer disagree with the overall conclusion [2] and are awaiting the decisions from international centers and health organizations. In the very recent article that was published in The Lancet Respiratory Medicine’s website by a large group of “PRIDE” consortium investigators that was funded by F Hoffmann-La Roche, the effectiveness of Tamiflu was revealed [3]. In this study, regardless of its aforementioned side effects, the importance of the neuraminidase inhibitor in the treatment of influenza A (H1N1) and the reduction in mortality rate due to influenza was discussed. Eventually, they concluded that oral Tamiflu (oseltamivir) might reduce mortality compared with no treatment. Regardless of this, the medicine is still prescribed (as tablets) to patients worldwide and is stocked until the purchased batches pass their expiry date [1]. It is worth noting that governmental departments such as the National Health Services (NHS) from the British government spent millions of Sterling Pounds on stockpiling the medicine in the case of a flu outcry [2]. Therefore, being risky is considered to be better than nothing if another viral pandemic suddenly becomes an epidemic, such as in the case of the corona virus [4,5].

However, this is not a morally correct solution to this very important issue.

Consequently, to answer the question in the title and bring up a solution to this, we insisted the urgency of finding a replacement that is safe and efficient for treating influenza which is the number one global killer, especially in children and the elderly. Flu is a nasty disease with new ones arising every few years. In 2006 the avian flu viruses (H5N1 and H9N1) outbreak arose, not long after, in 2009, the swine flu viruses (H1N1), and more recently in 2013 and early 2014, the Corona virus outbreak ensued and has already taken many lives in the Middle East [4,5], especially in Saudi Arabia and during the *Hajj* pilgrimage when millions of people are worshiping in a huge international close gathering.

In our most recent publication [6] we discussed the solution to viral infections by our idea of combining a synthetic medicine called Arbidol with a natural product called IMOD™ as a new medicine to increase their individual potency in a single dose formulation. In this paper, IMOD™ was introduced as a powerful natural medicine that can be effective against infectious diseases such as AIDS due to its interferonic action which generates lymphocytes and consequently increases CD4 cell counts [7,8], thereby improving life expectancy and quality of life for afflicted patients. This would be combined with Arbidol which is a synthetic medicine with good efficacy and no significant side effects, as discussed in several studies [9,10]. IMOD™ has been used within the last decade, the latter also for more than two decades. Their pharmacokinetics, safety, and efficacy were subjects to many publications. These have been implemented in several human clinical trials and influenza treatments for Arbidol. Their toxicity was very low in animal and human studies [11,12]. Therefore, these medicines are considered to be safe for human use and following years of prescription, no major issues have risen so far.

In comparison, mechanistically Tamiflu and Relenza (with generic name of zanamivir) are considered as neuraminidase inhibitors [1,3]. Tamiflu is administered orally as a tablet and the latter is administered by inhalation. The mechanisms of action for both medicines are very similar and bring about their effects by reacting with an enzyme that exists on the surface of the influenza virus [2]. In comparison to this, the Arbidol-IMOD combination has a different mechanism of action. In addition to Arbidol’s ability to react with the virus protein to disable the virus and prevent its penetration into cells, this combination is also distributed in the body quickly as an interferon inducer as well as possessing antioxidant activity, with essential elements such as selenium that boost the immune system further [7]. Therefore, the patient’s immune system prevents the body from becoming ill (prophylaxis of influenza) and it also fights viral diseases when the body needs it the most. However, the influence of IMOD™ in the treatment of influenza has not been discussed specifically anywhere in the literature, but the biological aspects of flavonoids in general has been the subject of many relevant studies [13,14]. The major classes of phytochemicals in IMOD™, including quercetin, rutin, apigenin, catechin, etc. have been reported to possess antiviral activity against some types of viruses. On top of this, they cause generation of lymphocytes and consequently increase CD4 cell counts [7,8] thereby improving life expectancy and quality of life of viral afflicted patients which shows the influence of IMOD™ in this preparation. In addition, different studies have shown that IMOD™ can increase interferon release from host cells significantly [8], thus regulating Interleukin-2 levels (IL-2) [15] and consequently increasing CD4 cell counts. One such interferon is interferon gamma (IFN-γ) which is crucial for innate and adaptive immunity. This is also the case with Arbidol, which also shows interferonogenic activity [10]. Therefore, the efficacy of IMOD™ in influenza was not directly the subject of any article, but by increasing the IL-2 levels shows it can fight infectious diseases. As a result, this provides strong evidence to indicate that IMOD™ can increase the efficacy of Arbidol further in the treatment of patients. Consequently, the new proposed idea is expected to produce a novel medicine that increases CD4 cell numbers, boosts the immune system further, and thus will be a better choice to prevent and cure viral infectious diseases in general.

It must be emphasized that chemically, Arbidol itself is a bromo-indol compound and in general, indols and especially the bromo-substituted ones are considered to be good sources against infectious viruses such as HIV and hepatitis B and C [16,17].

The toxicology studies of the novel medicine was carried out in mice by the investigators and showed practically zero toxicity for doses ranging from 2 g/kg to up to 4 g/Kg (unpublished data). Of course, a battery of genotoxicity and pharmacokinetic studies including drug interaction tests and related tests for immunosuppressive patients will be explored deeply for such a novel medicine in further studies.

The Authors believe with the new hypotheses and idea of the combination of Arbidol and IMOD™ [6], a stronger agent with a new formulation as discussed can bring a safe, effective medicine for preventing and curing infectious diseases such as different types of influenza, if the planned clinical trials and pharmacokinetic studies prove promising.

## References

[1] Jeferson T, Jones MA, Doshi P, DelMar CB, Hama R, Thompson MJ, Spencer EA, Onakpoya I, Mahtani KR, Nunan D, Howick J, Heneghan CJ: **Neuraminidase inhibitors for preventing and treating influenza in healthy adults and children.** *The Cochrane Collaboration, Wiley & Sons, Ltd* 2014, (4):24. www.thecochranelibrary.com. 10.1002/14651858.CD008965.pub4 PMC6464969 24718923

[2] Torjesen I: **Cochrane review questions effectiveness of neuraminidase inhibitors.** *BJM* 2014, **348**. g 2675/dio: 10.1136/bmj.g2675. 10.1136/bmj.g2675 24721619

[3] Muthuri SG, Venkatesan S, Myles PR, Leonardi-Bee J, Al Khuwaitir TSA, Al Mamun A, Anovadiya AP, Azziz-Baumgartner E, Báez C, Bassetti M, Beovic B, Bertisch B, Bonmarin I, Booy R, Borja-Aburto VH, Burgmann H, Cao B, Carratala J, Denholm JT, Dominguez SR, Duarte PAD, Dubnov-Raz G, Echavarria M, Fanella S, Gao Z, Gerardin P, Giannella M, Gubbels S, Herberg J, Iglesias ALH: **Effectiveness of neuraminidase inhibitors in reducing mortality in patients admitted to hospital with influenza A H1N1pdm09 virus infection: a meta-analysis of individual participant data.** 2014, www.thelancet.com/respiratory, 2014 http://dx.doi.org/10.1016/S2213-2600(14)70041-4.

[4] Zaki AM, Van Boheemen S, Bestebroer TM, Osterhaus AD, Fouchier RA (2012). Isolation of a novel corona virus from a man with pneumonia in Saudi Arabia. N Engl J Med.

[5] de Groot RJ, Baker SC, Baric RS, Brown CS, Drosten C, Enjuanes L, Fouchier RAM, Galiano M, Gorbalenya AE, Memish Z, Perlman S, Poon LLM, Snijder EJ, Stephens JM, Woo PCY, Zaki AM, Zambon M, Ziebuhr J (2013). Middle East respiratory syndrome corona virus (MERS-CoV); announcement of the corona virus study group. J Virol.

[6] Arastoo M, Khorram Khorshid HR, Radmanesh R, Gharibdoust F (2014). Combination of IMOD™ and Arbidol to increase their immunomodulatory effects as a novel medicine to prevent and cure influenza and some other infectious diseases. JMHI.

[7] Novitsky YA, Madani H, Gharibdoust F, Farhadi M, Farzamfar B, Mohraz M: *Use of a Combination of Ethanolic Rosa sp., Urtica Dioica and Tanacetum Vulgare Extracts, Further Compromising Selenium and Urea and Having Been Exposed to a Pulsed Electromagnetic Field, for the Preparation of a Medicament for Immune Stimulation and/or Treatment of HIV Infections.* USA: United States Patent Application; 2009. 20090208598.

[8] Khairandish P, Mohraz M, Farzamfar B, Abdollahi M, Shahhosseiny MH, Madani H, Sadeghi B, Heshmat R, Gharibdoust F, Khorram Khorshid HR (2009). Preclinical and phase 1 clinical safety of Setarud (IMOD™), a novel immunomodulator. Daru.

[9] Archakov AI, Guseva MK, Uchaykin VF, Ipatova OM, Dochshitsin YF, Medvedeva NV, Prozorovsky VN, Strekalova OS, Shironin AV (2012). Pharmaceutical Composition Containing Arbidol in the Form of Phospholipid Nanoparticles.

[10] Silin DS, Lyubomska OV, Ershov FI, Frolov VM, Kutsyna GA (2009). Synthetic and natural immune modulators acting as interferon inducers. Curr Pharm Des.

[11] Glushkov RG (1992). Arbidol Antiviral, immunostimulant, interferon inducer. Drug Future.

[12] Xin SU, Zenghua XIE, Yi SHI: **A randomized controlled clinical trial of arbidol in patients with acute viral upper respiratory infection.** *J Clin Int Med* 2005, **10**. http://en.cnki.com.cn/Article_en/CJFDTOTAL-LCLZ200510009.htm.

[13] Middleton E, Kandaswami C, Theoharides TC (2000). The effects of plant flavonoids on mammalian cells: implications for inflammation, heart disease, and cancer. Phrmacol rev.

[14] Selway JWT, Cody V, Middleton E, Harborne JB (1986). Antiviral Activity of Flavones and Flavans. Plant Flavonoids in Biology and Medicine: Biochemical, Pharmacological, and Structure-Activity Relationships.

[15] Mahmoodpoor A, Eslami K, Mojtahedzadeh M, Najafi A, Ahmadi A, Dehnadi-Moghadam A, Mohammadirad A, Baeeri M, Abdollahi M (2010). Examination of Setarud (IMOD™) in the management of patients with severe sepsis. Daru.

[16] Sharma V, Kumar P, Pathak D (2010). Biological importance of the Indole nucleus in recent years: a comprehensive review. J Heterocyclic Chem.

[17] Tetere Z, Kumpins V, Belyakov S, Zicane D, Turks M (2011). Synthesis and X-ray analysis of 7-bromoarbidol, an impurity standard of arbidol. J Heterocyclic Chem.

